# Ambulance deceleration causes increased intra cranial pressure in supine position: a prospective observational prove of principle study

**DOI:** 10.1186/s13049-021-00904-3

**Published:** 2021-06-30

**Authors:** Iscander M. Maissan, Boris Vlottes, Sanne Hoeks, Jan Bosch, Robert Jan Stolker, Dennis den Hartog

**Affiliations:** 1 Department of Anesthesiology, Dr. Molenwaterplein 40, 3015 GD Rotterdam, The Netherlands; 2 Regionale Ambulancevoorziening Hollands Midden, Research and Development, Vondellaan 43, 2332AA Leiden, The Netherlands; 3 Department of Trauma Surgery, Dr. Molenwaterplein 40, 3015 GD Rotterdam, The Netherlands

**Keywords:** Ambulance, Transportation, EMS, Traumatic brain injury, TBI, Intra cranial pressure, ICP, Optical nerve sheath diameter, ONSD, POCUS, Ultrasound

## Abstract

**Background:**

Ambulance drivers in the Netherlands are trained to drive as fluent as possible when transporting a head injured patient to the hospital. Acceleration and deceleration have the potential to create pressure changes in the head that may worsen outcome. Although the idea of fluid shift during braking causing intra cranial pressure (ICP) to rise is widely accepted, it lacks any scientific evidence. In this study we evaluated the effects of driving and deceleration during ambulance transportation on the intra cranial pressure in supine position and 30^°^ upright position.

**Methods:**

Participants were placed on the ambulance gurney in supine position. During driving and braking the optical nerve sheath diameter (ONSD) was measured with ultrasound. Because cerebro spinal fluid percolates in the optical nerve sheath when ICP rises, the diameter of this sheath will distend if ICP rises during braking of the ambulance. The same measurements were taken with the headrest in 30^°^ upright position.

**Results:**

Mean ONSD in 20 subjects in supine position increased from 4.80 (IQR 4.80–5.00) mm during normal transportation to 6.00 (IQR 5.75–6.40) mm (*p* < 0.001) during braking. ONSD’s increased in all subjects in supine position.

After raising the headrest of the gurney 30^°^ mean ONSD increased from 4.80 (IQR 4.67–5.02) mm during normal transportation to 4.90 (IQR 4.80–5.02) mm (*p* = 0.022) during braking. In 15 subjects (75%) there was no change in ONSD at all.

**Conclusions:**

ONSD and thereby ICP increases during deceleration of a transporting vehicle in participants in supine position. Raising the headrest of the gurney to 30 degrees reduces the effect of breaking on ICP.

## Background

Traumatic brain injury (TBI) is the leading causes of death in young people [1]. It’s incidence is 262/100.000 people and is responsible for 10.5 deaths per 100.000 inhabitants in Europe [2]. After primary brain injury occurs, hypo-perfusion of the brain and low oxygen saturation of the blood can cause secondary brain injury [3]. Hypo-perfusion of brain tissue can be caused by low systemic blood pressure, but more often by an increased intracranial pressure (ICP) that compromises cerebral blood flow [3, 4]. Cerebral perfusion pressure (CPP) is defined as the difference between mean arterial pressure (MAP) and intracranial pressure (ICP). There is a relationship between the time of low CPP’s and the extent of secondary brain injury [5]. Autoregulation of cerebral perfusion is often impaired or absent after head injury which makes the brain more vulnerable to pressure changes [3, 4]. An increase in ICP during an ambulance ride could compromise cerebral perfusion and induce ischemia of the brain, further increasing the risk of secondary brain injury [3].

In the Dutch prehospital care system ambulances are manned by a medically trained driver and a specialized nurse that can provide advanced life support to most patients and circumstances. Physician staffed mobile medical teams can be dispatched to assist ambulance crews in high complex cases. When traumatic brain injury has occurred these teams are utilized to start anaesthetic brain protective therapy in the field. Anaesthesia decreases the oxygen consumption of the brain and lowers the ICP [6]. Hypertonic saline may decrease brain tissue swelling and elevating the headrest results in better drainage of venous blood from the head trough gravitational forces [7–9]. Head-injured patients should primarily be admitted to a level 1 trauma center for better neurological outcomes [5]. These level 1 trauma centres are widespread across the country and may therefore lead to longer transportation times. Based on the hypothesis that fluid shifts increases ICP ambulance drivers in Netherlands are trained to drive as smoothly as possible [10, 11]. The hypothesis states that fluid shifts have the potential to increase the ICP during deceleration of the moving ambulance due to increased venous stasis and backflow in the head. A similar mechanism has been proven to increase the ICP and optical nerve sheath diameter (ONSD) in Trendelenburg position and after application of a rigid cervical collar [12, 13].

The ONSD is in continuum with the intracranial cavity and cerebro spinal fluid percolates freely in the optical nerve sheath. When the ICP rises the diameter of this sheath will distend [14, 15]. To our knowledge there is no scientific evidence on the effects of deceleration on the ICP in trauma patients. The objective of this study is to examine this in healthy subjects with sonographic ONSD measurement. We hypothesize that raising the headrest of the gurney 30° upwards will decrease ICP during deceleration. The aim of the study is to provide knowledge on how to safer transport TBI patients.

## Methods

This observational study was set up as a prospective proof of principle study. Twenty participants were recruited from the regional ambulance service Hollands Midden. The participants were aged over 17 years and didn’t have any self-reported ocular, intracranial disease or vaso-active medication. The medical ethical commission of the Erasmus Medical Center in Rotterdam waived a full review according to the Dutch Medical Research with Human Subjects Law. (MEC2018–1377) All participants were employees of the ambulance service and responded to a written invitation on the intranet of the company and gave their written informed consent.

The experiment was carried out on all participants on the 9th of July 2019 between 8:00 am and 5:00 pm.

**Fig. 1:**
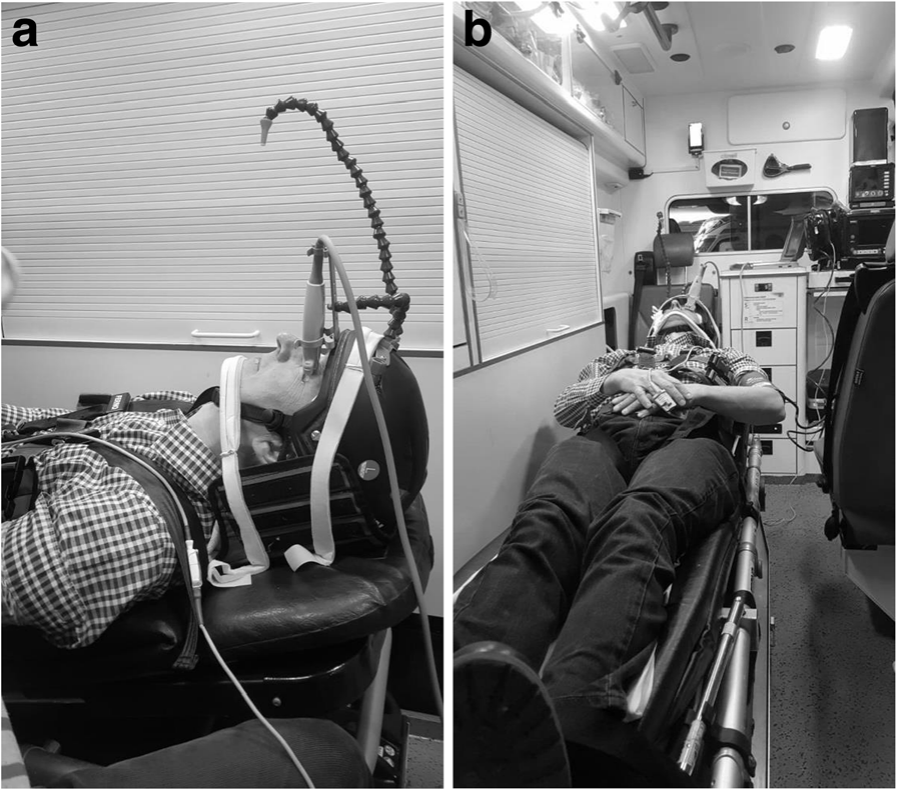
**a** and **b**. Research setup in the back of the ambulance with the two mounts on the helmet. One to hold the transducer and the other to create a focus point for the non-measured eye to prevent from moving the eyes during braking

To perform measurements during ambulance driving we used a regular ambulance (Mercedes Sprinter 419 cdi bluetec. 190 PK, Stuttgart, Germany) in which participants were transported in head first supine position on a gurney. Participants were axially fixated with a Kendrick Extrication Device (KED from Ferno-Washington Inc. Ohio, USA). They were placed in supine position inside the ambulance and were fitted with a bicycle helmet which contained two mounts. (Fig. 1a,b) The helmet was adjustable to account for different sizes of head circumference. (58–61 cm / 22 ¾ - 24 in.). The first mount held the probe of the ultrasound machine (M-Turbo with 7.5 MHz linear probe; ocular setting, mechanical index = 0.2: FUJIFILM SonoSite Inc., Bothell, Washington, USA). This mount made it possible to visualize and freeze the picture of the ONSD during deceleration and measure the diameter direct afterwards. The second mount held an extended arm with an orange dot at the end for the contralateral eye to focus on during deceleration. The helmet was fixated to the KED as a non-slidable whole to prevent the helmet from moving on the head during deceleration. Both the helmet and the KED were applied to the volunteer as described in the user’s manual of the devices.

A baseline ONSD measurement without acceleration and deceleration was performed for all participants. For the second measurement the ambulance steadily accelerated to a speed of 50 km per hour (km/h) and subsequently decelerated to a speed of 0 km/h on a 10 m distance track. During deceleration the freeze button was pressed and the ONSD was measured with the ultrasound probe on the left eye by an experienced sonographer [IM]. The examiner was seated at the ambulance chair on the head side of the gurney. The third and fourth measurements were conducted in identical approach, however with the headrest of the gurney raised 30° upwards. A digital leveller was used to measure the angle. (Bubble Level PRO by Gamma Play on an Samsung Galaxy S8 Android Device) All participants had their blood pressure, pulse rate and oxygen saturation measured during the experiment with the standard ambulance monitor (Tempus ALS, RDT/Philips Healthcare, Amsterdam, The Netherlands).

For this experiment the ambulance was driven by an experienced well trained ambulance driver. All experiments were taken on the same driving school training circuit. (Bremmer transportcollege, Hazerswoude, the Netherlands).

Participant characteristics are described as number (percentage) or mean (standard deviation) / median (interquartile range). Normal distribution of the data was assessed by visual inspection of histogram and Q-Q plots and/or normality test. Difference in ONSD between the conditions were analysed using the Wilcoxon signed-rank test because results were not normally distributed. We considered the ONSD to be clinically relevant changed if the ONSD differed > 0,2 mm from the baseline measurement since the intra-observer variability has been reported to be 0.2 mm in previous literature [14].

## Results

**Fig. 2:**
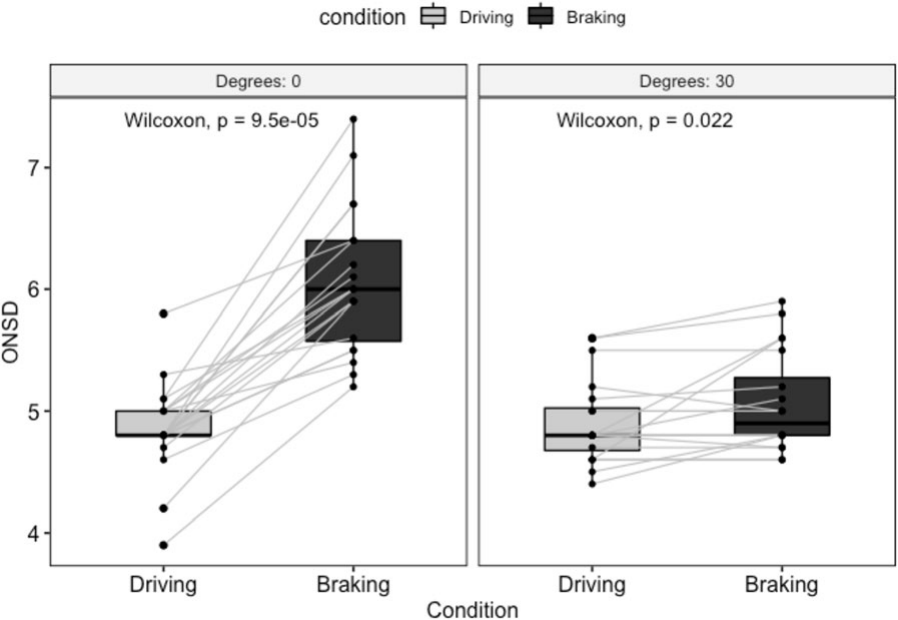
ONSD during driving and braking of the ambulance in 0 degrees and 30 degrees head up position

We included 20 healthy participants after they had given their written informed consent. Participant characteristics are shown in Table 1. Vital parameters such as systolic blood pressure, diastolic blood pressure, pulse and pulse oximetry were all considered in normal range both before and after testing. The ONSD measurements are shown in Fig. 2. With the gurney at 0 degrees the ONSD during baseline and braking measurements was 4.80 (IQR 4.80–5.00) mm and 6.00 (IQR 5.75–6.40) mm respectively (*p* < 0.001). Raising the gurney to 30 degrees resulted in an ONSD baseline and during braking measurements of 4.80 (IQR 4.67–5.02) mm and 4.90 (IQR 4.80–5.02) mm respectively (*p* = 0.022). Overall, a 24% increase in ONSD during braking with the gurney at 0 degrees and a 0% increase in ONSD during braking with the gurney at 30 degrees was observed. All 20 participants with the gurney at 0 degrees showed > 0.2 mm increase in ONSD during braking, whilst with the gurney at 30 degrees 5 participants showed > 0.2 mm increase in ONSD during braking.

**Table 1:** subject characteristics

Variable	Value (SD)
No. of subjects	20
Age in years	40 (10)
No. of males (%)	15 (75%)
Baseline oxygen saturation	97 (1)
Post-test oxygen saturation	96 (1)
Baseline systolic blood pressure	132 (10)
Post-test systolic blood pressure	132 (10)
Baseline diastolic blood pressure	81 (10)
Post-test diastolic blood pressure	82 (11)
Baseline heart rate	71 (10)
Post-test heart rate	71 (8)

## Discussion

In this study we investigated the relationship between the fluid shifts during ambulance braking and ICP increases. The results demonstrate that the deceleration force of braking increases the ONSD and hence rises ICP during ambulance transportation in supine position.

Elevation of the headrest (30 degrees) is part of the ICU protocol for patients with persisting elevated ICP’s (> 20 mmHg) to drain superfluous venous blood from the head [5].

Today’s prehospital transportation protocols in the Netherlands prescribe immobilisation in supine position [9, 10]. Based on our results we may reduce secondary brain injury by raising the headrest as soon as possible in pre hospital care if systemic blood pressure is in normal or supra-physiological range. One can argue that raising the headrest may worsen neurological outcome of the lower extremities if lumbar spinal injury is present. Although there is a risk of iatrogenic paralyzes of the legs we consider life should be treated before limb if severe TBI is suspected. To evaluate the effects of an elevated headrest on ONSD changes in a decelerating ambulance we used ultrasound to measure the sheath that surrounds the optical nerve behind the eye. This sheath is in a continuum with the meninges around the brain. Cerebro-spinal fluid percolates freely from the intra cranial cavitum into the sheath. This results in a sheath distention when the intra cranial pressure rises [15]. This method has shown a reliable diagnostic accuracy (sensitivity 0.90 [CI 0.80–0.95,] specificity 0.85 [CI 0.73–0.93]) for detecting a raised intracranial pressure [16, 17]. Previously our group reported that sonographic measurement of the ONSD can be used as a quick and non-invasive monitor of changes in intracranial pressure in one individual (R^2^ = 0,80) [14]. In this study we took several measurements in different circumstances in the same volunteer including a baseline measurement to evaluate possible changes in ONSD during transportation. Although the correlation between ICP and ONSD seems to be strong in repetitive measurements the clinical consequences aren’t clear. ONSD response to pressure changes seems to be a linear correlation but depends strongly on the elasticity of ones sheath [15]. Differences in baseline ONSD’s have been reported in different sexes and ethnic groups [18]. The cut-off point of the ONSD representing ICP ≥ 20 mmHg is still under debate. This means that we can’t calculate ICP’s from ONSD’s and we should considered the ONSD’s to be rather qualitative than quantitative data [14].

Although our testing setup is as much standardized as it could get, the forces may have been slightly different in the repeated deceleration measurements.

Because measurements were taken during transportation the examiner could not be blinded to the intervention of braking. This may have introduced an observer bias in this study. Although we think fluid shift leads to an increase in ICP and ONSD it may as well be increased due to anxiety and breath holding during braking [2, 19]. This effect seems to minor as in upright position the increase in ICP is diminished largely but can explain the significant rise in ONSD in elevated position.

After traumatic brain injury has occurred time is of the essence to prevent secondary brain injury to extend and worsen patient outcome*.* Based on the findings in this experiment we suggest to revise the current transportation protocols and elevate the headrest of the ambulance gurney as soon as feasible when transporting brain injured patients.

## Conclusions

ONSD and thereby ICP increases during deceleration of an transporting vehicle in patients in supine position. Raising the headrest of the gurney to 30 degrees reduces the effect of breaking on ICP. More research is needed on this subject with varying driving speeds, varying headrest angles and eventually with actual TBI patients during transportation.

## Data Availability

The datasets used and/or analysed during the current study are available from the corresponding author on reasonable request.

## Abbreviations

ICP: Intra cranial pressure
ONSD: Optical nerve sheath diameter
CPP: Cerebral perfusion pressure
MAP: Mean arterial pressure
TBI: Traumatic brain injury
PK: Horse power
KED: Kendrick extrication device

## Declarations

### Concent for publication

Consent to publish the pictures of Jan Bosch is given in a written informed consent and uploaded separately from this manuscript.
