# Correction to: Ambulance deceleration causes increased intra cranial pressure in supine position: a prospective observational proof of principle study

**DOI:** 10.1186/s13049-021-00926-x

**Published:** 2021-07-31

**Authors:** Iscander M. Maissan, Boris Vlottes, Sanne Hoeks, Jan Bosch, Robert Jan Stolker, Dennis den Hartog

**Affiliations:** 1 Department of Anesthesiology, Dr. Molenwaterplein 40, 3015, GD Rotterdam, The Netherlands; 2 Regionale Ambulancevoorziening Hollands Midden, Research and Development, Vondellaan 43, 2332AA Leiden, The Netherlands; 3 Department of Trauma Surgery, Dr. Molenwaterplein 40, 3015, GD Rotterdam, The Netherlands

**Correction to: Scand J Trauma Resusc Emerg Med 29, 87 (2021)**

**https://doi.org/10.1186/s13049-021-00904-3**

Following the publication of the original article [1], the authors informed as of an error in the title. The word “prove” of the original title has been changed to “proof”.

The article title now reads: “Ambulance deceleration causes increased intra cranial pressure in supine position: a prospective observational proof of principle study”.

The correct title is shown in this Correction, as well as the original article which has already been updated online.
